# Increased presence of oxidized low‐density lipoprotein in the left ventricular blood of subjects with cardiovascular disease

**DOI:** 10.14814/phy2.12726

**Published:** 2016-03-31

**Authors:** Chandrakala Aluganti Narasimhulu, Dmitry Litvinov, Bhaswati Sengupta, Danielle Jones, Chittoor Sai‐Sudhakar, Michael Firstenberg, Benjamin Sun, Sampath Parthasarathy

**Affiliations:** ^1^ Burnett School of Biomedical SciencesCollege of MedicineUniversity of Central FloridaOrlandoFlorida; ^2^ Division of Cardiothoracic SurgeryThe Ohio State University Medical CenterColumbusOhio; ^3^ Scott and White HealthTempleTexas; ^4^ Department of SurgeryNorth East Ohio Medical UniversityAkronOhio; ^5^ Minneapolis Heart InstituteMinneapolisMinnesota

**Keywords:** Cholesterol efflux, ejection fraction, foam cells, heart failure, paraoxonase

## Abstract

Oxidized LDL (Ox‐LDL) and oxidative stress have been implicated in both atherosclerosis and congestive heart failure (HF) development. Here, we tested whether Ox‐LDL levels in left ventricular blood (LVB) might differ from those of venous peripheral blood (PB), and whether the level might depend on cardiac function. We also tested whether the LDL molecule is likely to have a longer residence time in the left ventricle of HF subjects with low ejection fraction (EF). The aim of this study was to determine Ox‐LDL levels, paraoxonase 1 (PON1) activity, and cholesterol efflux capacity (CEC) of PB and LVB, and correlate these values with LVEF. Sixty‐one HF patients underwent preoperative transthoracic echocardiographic assessment of ventricular function. LVEFs were determined using Simpson's biplane technique. LVB and PB levels of Ox‐LDL were determined, and PON1 activity and plasma cholesterol efflux capacity were measured. A significant increase in the levels of Ox‐LDL in LVB was noted as compared to levels in PB, even when EF was near normal. However, as ejection fraction decreased, the level of Ox‐LDL in PB approached that of the LVB. PON1 activity and cholesterol efflux studies indicated increased oxidative stress in LVB and a decreased ability to promote cholesterol efflux from lipid‐enriched macrophages. The results suggest that LVB is more oxidatively stressed compared to PB, and therefore LV tissue might be affected differently than peripheral tissues. We recently reported that brain natriuretic peptide (BNP), a marker for HF, is induced by Ox‐LDL, so it is possible localized factors within the LV could profoundly affect markers of HF.

## Introduction

Oxidative stress plays a significant role in the pathogenesis of several cardiovascular diseases, including heart failure (HF) (Griendling and Fitzgerald 2003; Madamanchi et al. 2005; Mueller et al. 2005). It has been noticed that several plasma biochemical markers of oxidative stress were increased in HF subjects (Belch et al. 1991; McMurray et al. 1993; Keith et al. 1998), suggesting the importance of oxidative stress mechanisms in congestive HF progression. Atherosclerosis is one of the major risk factors for HF. Oxidized low‐density lipoprotein (Ox‐LDL) were suggested to play a key role in the development of atherosclerosis (Steinberg 1997). Plasma levels of Ox‐LDL are considered to be a prognostic indicator of mortality in subjects with congestive HF (Tsutsui et al. 2002). Increased levels of Ox‐LDL and Ox‐LDL antibodies in the plasma are correlated with a lower EF, increased severity of clinical symptoms (Tsutsui et al. 2002; George et al. 2006), and increased postinfarction left ventricular (LV) volumes (Fuji et al. 2002).

Serum PON1 is synthesized mainly by the liver and circulates in association with apolipoprotein A‐I (APOAI) and high‐density lipoprotein (HDL) (Teiber et al. 2004). PON1 is known to hydrolyze various types of substrates, such as arylesters, phosphate esters, and lactones (Teiber et al. 2003), including homocysteine thiolactones (Jakubowski 2000). More significantly, PON1 is known to inactivate lipid peroxides and hydrogen peroxide and therefore is known to offer protection against oxidative stress (Aviram et al. 1998; Shamir et al. 2005; Deakin et al. 2007). In addition, PON1 has been shown to inhibit the oxidation of LDL, a key step in atherosclerosis (Abbott et al. 1995). It also protects against homocystinylation, a posttranslational modification of proteins which attenuates the biological activity of the protein (Duell and Malinow 1997). In addition, the antiatherosclerotic effects of PON1 are supposed by its lactonase activity and its role in reverse cholesterol transport (RCT) (Shih et al. 1996).

In this study, we tested the hypothesis that Ox‐LDL levels would be increased in the left ventricular blood of HF patients as compared to their respective levels in the peripheral blood. Increased Ox‐LDL levels correlated with low ejection fraction in LVB, decreased PON1 activity and reduced RCT. We report that the levels of Ox‐LDL in the peripheral blood increased with decreasing EF, approaching levels of Ox‐LDL noted in the LVB.

## Materials and methods

### Chemicals

Human Ox‐LDL ELISA kit (10‐1143‐01) from Mercodia Inc. (Winston Salem, NC) was purchased and used. RPMI 1640, fetal bovine serum (FBS), sodium pyruvate, L‐glutamine, penicillin/streptomycin (PS), 1X phosphate‐buffered saline (PBS), and NBD‐cholesterol were purchased from Invitrogen Life Technologies (Carlsbad, CA). Cholesterol, lysophosphatidylcholine (LysoPC) from egg yolk, oleic acid, and other routine chemicals were purchased from Sigma (St. Louis, MO). [1, 2‐^3^H (N)] cholesterol, and [1‐^14^C] Oleic acid were purchased from American Radiolabeled Chemicals (St. Louis, MO).

### Human patients

Following The Ohio State University School of Medicine Institutional Review Board (IRB) approval, 62 patients (with consent) undergoing routine cardiovascular surgery [coronary artery bypass grafting (CABG), valve repair/replacement (AVR, MVR, TVR: aortic, mitral, or tricuspid valve replacements, respectively) or other open heart surgeries] at The Ohio State University Medical Center were enrolled and categorized as shown in Table 1. The HF patients enrolled in this study were grouped via New York Heart Association (NYHA) functional classification as Class II HF. Though many of the subjects had preserved ejection fractions, they were diagnosed with heart failure due to diastolic dysfunction. All subjects underwent preoperative transthoracic echocardiographic assessment of ventricular function. Left ventricular ejection fractions were determined using the Simpson's bi‐plane technique, and standard as well as considered US measurements represented in Table S1. These results were validated with routine intraoperative assessment. Two milliliter of LVB was sampled by aspirating blood using a 23‐gauge needle and 10 cc syringe placed through the ventricular apex. Simultaneously, 5 mL of PB was sampled from a peripheral venous line. Blood was placed in heparinized tubes and labeled with a patient number. Plasma was separated immediately after collection and stored at −80°C for further analysis. No patients had clinical evidence of active ischemia at the time of blood sampling.

**Table 1:** Patients grouped by surgery and EF%

EF%^a^	Number (M + F)	Type of surgery and no. of patients (M + F)		
		CABG	Aortic valve repair	CABG + Valve repair
>60%	20 (11 + 9)	15 (10 + 5)	3 (1 + 2)	2 (0 + 2)
>40% & <60%	28 (23 + 5)	13 (12 + 1)	8 (6 + 2)	7 (5 + 2)
<40%	13 (8 + 5)	5 (4 + 1)	3 (1 + 2)	5 (3 + 2)
Total	61 (42 + 19)	33 (26 + 7)	14 (8 + 6)	14 (8 + 6)

CABG, Coronary artery bypass grafting; M, Male; F, Female.

a EF% of one patient was not reported.

### Selection criteria

All subjects were optimized with medical management prior to surgery, and these included the use of beta‐blockers, angiotensin‐converting enzyme (ACE) inhibitors, statins, and diuretic therapy. The use of such therapies themselves could affect Ox‐LDL production. Our main objective was to demonstrate increased levels of Ox‐LDL in the LV cavity of HF subjects, and therefore, for each patient the PB sample serves as the control. For this reason, comparison of our patient population with a control population is not feasible. As per clinical criteria, we considered only HF subjects less than 85 years old with an EF less than or equal to 70%.

### Determination of Ox‐LDL levels

Lipoprotein measurement is a major diagnostic tool to determine the risk of atherosclerosis and other cardiovascular diseases (CVDs). Analyzing plasma Ox‐LDL levels is essential in order to identify disease and for effective treatment. Mercodia Ox‐LDL ELISA kit is useful for in vitro quantitative measurement of Ox‐LDL in human blood serum or plasma, and according to the supplier, has an intra‐assay coefficient of variation (CV) of <6% and interassay CV of <7%. Plasma sample collection, storage, and processing were conducted via the manufacturer's protocol (Mercodia Inc., NC). According to Perman et al. (Perman et al. 2004), storage of blood samples at −80°C for 3 years does not have any significant effect on absolute levels of Ox‐LDL in human plasma. All samples were processed uniformly. Both LVB and PB plasma samples from each subject were analyzed within one assay plate to ensure equal ex vivo conditions. Thus, should any ex vivo oxidation occur, it would be similar for intrasubject samples and standards.

Twenty‐five microliter of human plasma was analyzed using a sandwich ELISA kit (Mercodia Inc., Winston Salem, NC), a solid phase two‐site enzyme immunoassay, in which two monoclonal antibodies are directed against separate antigenic determinants of oxidized apolipoprotein B_100_ molecule. One of the antibodies binds to the surface of a 96‐well plate, and the other antibody, conjugated with peroxidase, binds in the solution. After incubation, the bound conjugate was detected by reaction with 3, 3′, 5, 5′‐tetramethylbenzidine (TMB). The reaction was stopped by the addition of 0.5 mol/L H_2_SO_4_ and absorbance was measured at 450 nm using a microplate reader (Bio‐Rad, Hercules, CA). Results were analyzed using Graphpad Prism 5 (GraphPad Software, La Jolla, CA).

### Determination of PON 1 activity

Plasma PON1 arylesterase activity was measured by following the method of Abbott et al. (Abbott et al. 1995). Briefly, 5 *μ*L of human plasma was incubated with p‐nitrophenyl acetate in 200 *μ*L phosphate buffer with 2 mmol/L CaCl_2_ and MgCl_2_ at 37°C. The conversion rate of p‐nitrophenyl acetate into p‐nitrophenol was determined by changes in the optical absorption at 410 nm. Readings were recorded at the end of 5 min.

### Lipid profile

Blood lipid profile measurements of total cholesterol (TC), triglycerides (TRG), HDL‐cholesterol, and LDL‐cholesterol were determined by using the Cholestech L*D*X Analyzer (Cholestech Corp, Hayward, CA). According to National Cholesterol Education Program‐Adult Treatment Panel III (NCEP‐ATP III) guidelines, abnormal serum cholesterol values are defined as (1) TC ≥ 200 mg/dL; (2) TRG ≥ 150 mg/dL; (3) HDL‐C < 40 mg/dL; and (4) LDL‐C ≥ 100 mg/dL (optimal) and 130 mg/dL (borderline high). According to these guidelines, the CV for measurement of precision and accuracy for TC is <3%, TG is <5%, and for both HDL and LDL is <3%. A comparison study by Dale et al. showed that the Cholestech L*D*X is the only lipid analyzer able to produce a full lipid panel with accuracy comparable to that of the gold standard, ultracentrifugation (Dale et al. 2008). Several other studies also corroborate this comparable accuracy (Bard et al. 1997; Volles et al. 1998; Panz et al. 2005; Shemesh et al. 2006).

### Analysis of cardiac troponin I levels

Levels of cardiac troponin I in all blood samples were measured by using the ADVIA Centaur XP Immunoassay System (Global Siemens Healthcare Sector, Germany).

### Cell culture

RAW 264.7 cells were obtained from American Type Culture Collection (ATCC) (Manassas, VA). Cells were grown as a monolayer in flasks and maintained in RPMI 1640 medium supplemented with 10% fetal bovine serum, 2 mM L‐glutamine, and 1× penicillin/streptomycin antibiotic solution. Cultures were maintained in a 5% CO_2_ atmosphere at 37°C. For experiments involving foam cell development, cells were incubated in basal medium with 0.1% lipoprotein deficient serum (LPDS).

### Foam cell preparation for cholesterol efflux

Fluorescent cholesterol and radiolabeled cholesterol were prepared as described previously (Sengupta et al. 2013). Foam cells containing fluorescent derivative of cholesterol (NBD) or radiolabeled cholesterol were prepared as described previously (Sengupta et al. 2013). Briefly, RAW 264.7 macrophages were seeded in a 48‐well plate at a concentration of 1.2 × 10^6^ cells/ml to gain 65–70% confluence overnight. Cells were washed with warm sterile PBS once, followed by 4 h of incubation in RPMI 1640 containing 0.1% LPDS. After 4 h, these cells were incubated with 40 *μ*L of cholesterol or cholesteryl ester (CE) (NBD or ^3^H‐cholesterol)/LysoPC mixed micelles for 16–18 h in the presence of 40 μmol/L oleic acid. For quantitative studies with radioactive cholesterol, NBD‐cholesterol was substituted with ^3^H‐cholesterol to give a final specific radioactivity of 5000 DPM/nmol of cholesterol.

### Incubation of foam cells with HF patient plasma samples for cholesterol efflux studies

For this study, plasma samples from both LV (12 + 12 samples) and PB (12 + 12 samples) of HF subjects with higher EF (>60%) and lower EF (<45%) were considered. RAW 264.7 macrophages were incubated either with fluorescent or tritiated cholesterol/LysoPC mixed micelles overnight, then washed with PBS twice. Cells were then incubated with 5 *μ*L of patient plasma samples for 4 h in 500 *μ*L of Hanks Balanced Salt Solution (HBSS). At the end of incubation, samples were collected and efflux of labeled cholesterol was measured. For quantitative studies of cholesterol efflux, foam cells were developed using ^3^H‐cholesterol (and unlabeled cholesterol)/LysoPC mixed micelles as described previously (Sengupta et al. 2013). Efflux of ^3^H‐cholesterol in HBSS upon incubation with patient plasma samples was measured by liquid Microbeta 2 scintillation counter (Perkin Elmer, Waltham, MA). HBSS alone without patient plasma, incubated with foam cells, served as a background value in cholesterol efflux experiments.

### Deproteination of samples

HBSS and plasma samples used for cholesterol efflux studies involving NBD cholesterol were not suitable for direct fluorescence measurement due to their interference with protein. To overcome this, samples were subjected to deproteination by precipitating the protein with one ml of ice‐cold acetone and isopropyl alcohol (1:1 v/v) for 30 min at 4°C followed by centrifugation at 400 × *g* for 10 min. The organic solvent phase was evaporated under a stream of nitrogen, after which 100 *μ*L aliquots of the supernatants were taken and fluorescence was measured using a fluorescence plate reader (Envision 2014 Multilabel plate reader, Perkin Elmer, Waltham, MA). Fluorescence of effluxed NBD‐cholesterol was measured at emission spectra of 535 nm upon excitation at 485 nm.

### Statistical analysis

For the detection of difference between Ox‐LDL levels in LVB and PB samples, the number of participants was adequate in power to detect significant changes. Calculations for sample size with appropriate statistical power were performed using G*Power 3.1.2 software (Dusseldorf, Germany). Retrospective power analysis for differences between Ox‐LDL levels in PB and LVB revealed statistical power of 0.03, assuming 0.05 alpha error probability level (*P*‐value) in two‐tail matched‐pair *t*‐test. Power of the sample size was calculated using PAWE software (Gordon et al. 2002). The relationship between Ox‐LDL levels in LVB or PB and EF was analyzed by Pearson correlation. Correlation coefficients and two‐tailed *P*‐values were calculated using Prism 5 for Windows (GraphPad Software, La Jolla, CA). *P* values for differences between EF groups were calculated using one‐way analysis of variance (ANOVA) and subjected to Bonferroni multiple comparison tests and considered significant when *P* < 0.05, using Prism 5 for Windows.

## Results

### Increased Ox‐LDL levels in LVB of HF subjects

**Figure 1:**
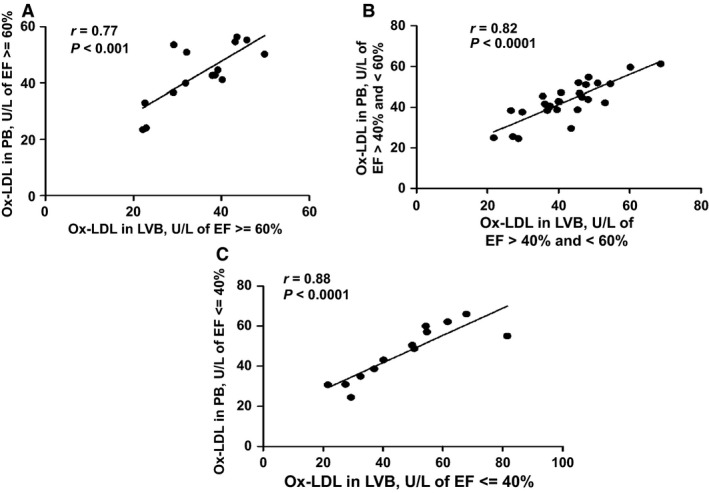
Increased Ox‐LDL levels in LVB and PB of HF subjects. Following Institutional Review Board (IRB) approval, 62 subjects (consented) undergoing routine cardiovascular surgery [coronary artery bypass grafting (CABG), valve repair/replacement (AVR, MVR, TVR: aortic, mitral, or tricuspid valve replacements, respectively) or other open heart surgeries] at The Ohio State University Medical Center were enrolled. The subjects enrolled in this study were NYHA class II. All subjects underwent pre‐operative transthoracic echocardiographic assessment of ventricular function. Left ventricular ejection fractions were determined using the Simpsons bi‐plane technique. LVB and PB samples were collected. Sixty‐one HF subjects samples were analyzed for the presence of Ox‐LDL levels by Mercodia ELISA kit. Correlation between Ox‐LDL levels in LVB and PB (A) ≥60% (B) >40% and <60% (C) ≤40%. Pearson Correlation *P*‐value as well as *r* values are shown. All the samples were analyzed in triplicates and values are expressed as mean ± SD. *P <* *0.05.

Ox‐LDL levels were compared in the LVB and PB of HF patients. A significant difference in Ox‐LDL levels was observed in LV blood as compared to PB (*P *=* *0.031), particularly in patients with EF ≥ 60%. Increased Ox‐LDL levels were also found in the LV blood of subjects with lower EF, although the levels differed less between LVB and PB in this circumstance. Thus, as the EF lowers to ≤40%, the peripheral blood Ox‐LDL levels approach those of the LVB. The difference in Ox‐LDL levels in PB between EF ≥ 60% group and EF >40% and <60% group was significant. The correlations between Ox‐LDL levels in LVB and PB have been represented in Figure 1.

**Figure 2:**
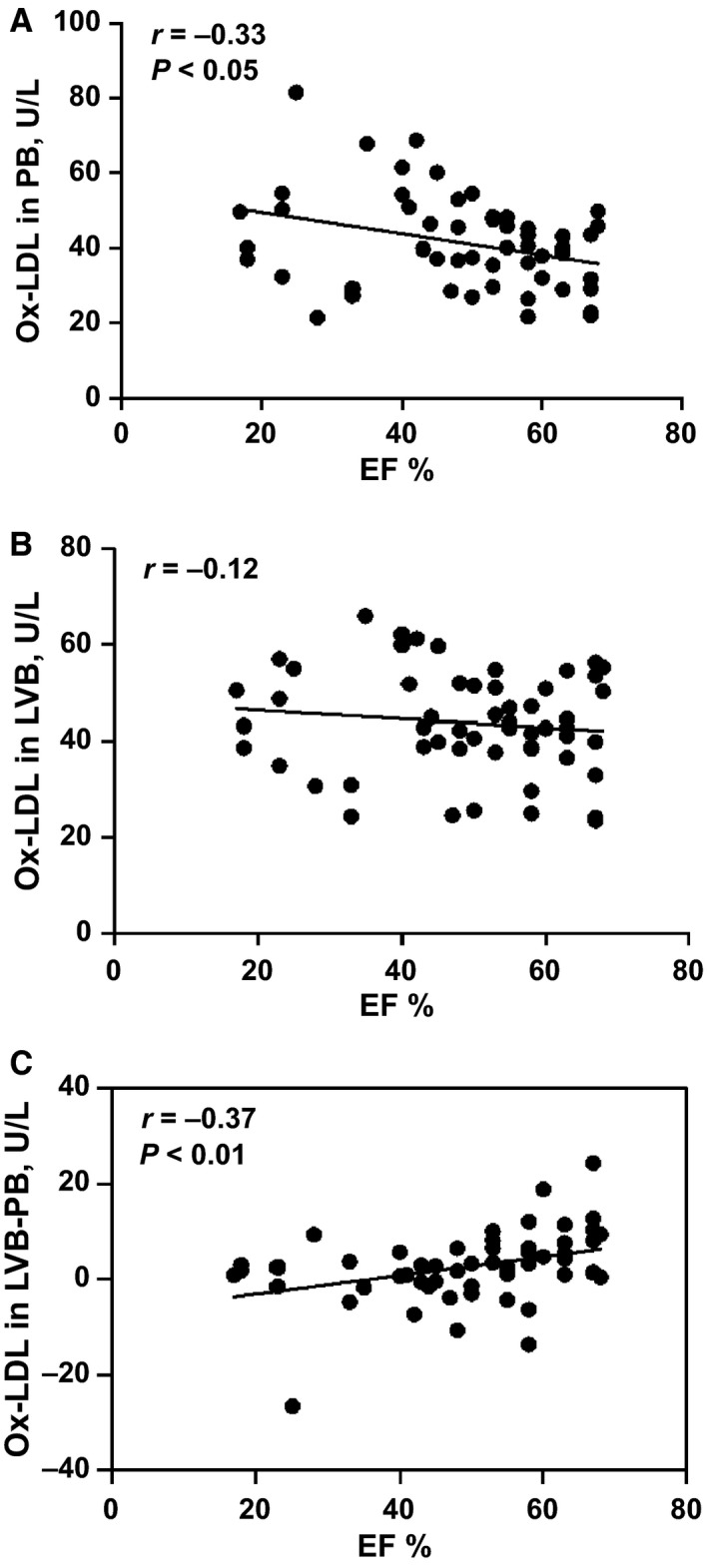
Correlation between Ox‐LDL in PB/LVB versus EF: Correlation between Ox‐LDL in PB (A), LVB (B), and LVB‐PB (C) with %EF. The correlation between Ox‐LDL level and EF% was negative for both PB and LVB, however, statistically significant correlation was observed for PB only (Pearson correlation *P* < 0.05). The difference between Ox‐LDL level in LVB and PB increased with the increase of EF%.

The correlation between Ox‐LDL level and EF% was negative for both PB and LVB (Fig. 2A and B); however, statistical significance of the correlation was observed only for PB (Pearson Correlation *P* < 0.05) (Fig. 2A). The difference between Ox‐LDL levels in LVB and PB significantly increased with increasing EF% (Fig. 2C).

### Decreased PON1 activity in LV blood of HF subjects

**Figure 3:**
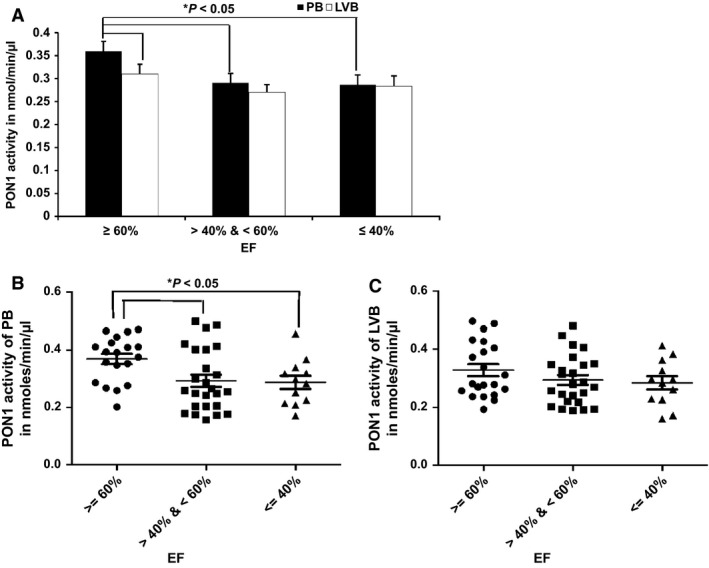
Decreased PON1 levels in LV blood of HF subjects. Following Institutional Review Board (IRB) approval, 62 subjects (consented) undergoing routine cardiovascular surgery [coronary artery bypass grafting (CABG), valve repair/replacement (AVR, MVR, TVR: aortic, mitral, or tricuspid valve replacements, respectively) or other open heart surgeries] at The Ohio State University Medical Center were enrolled. The subjects enrolled in this study were NYHA Class II. All subjects underwent preoperative transthoracic echocardiographic assessment of ventricular function. Left ventricular ejection fractions were determined using the Simpsons bi‐plane technique. LVB and PB samples were collected. Sixty‐one HF subjects samples were analyzed for the presence of PON1 levels as described in the methods. (A) There was a statistically significant difference in PON1 activity levels in LVB and PB of subjects having EF ≥ 60% as evaluated by paired two‐tailed Student *t* test (*P <* *0.05). (B) PON1 activity of PB versus EF C) PON1 activity of LVB versus EF. All the samples were analyzed in triplicates and values are expressed as mean ± SD.

Levels of PON 1 activity were compared in LVB and PB of HF patients. As shown in Figure 3, PON1 activity was decreased both in LVB as well as in PB with decreasing EF. It was observed that there was a statistically significant difference in PON1 activity between LVB and PB in patients with EF ≥ 60% (*P *<* *0.05). Surprisingly, as the EF decreases, the difference between PON1 activity in PB and LVB is minimal, though both activities are reduced as compared to higher EF%.

### Blood lipid profiles

**Figure 4:**
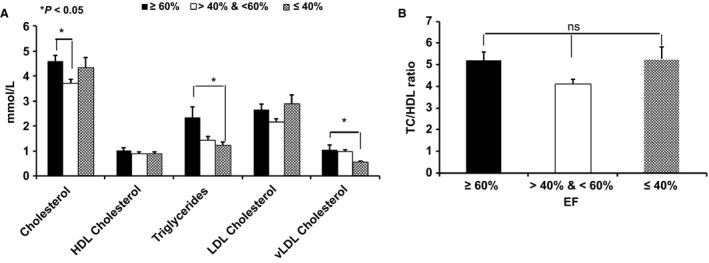
Blood lipid profiles of HF patients: (A) Blood lipid profiles (B) TC/HDL ratio. There was a significant decrease in triglycerides as the EF decreases, whereas LDL‐C levels were increased as EF decreases. No significant change was observed in HDL‐C levels (*P <* *0.05). (One‐way ANOVA with Bonferroni's multiple comparison test).

The EF of patients ranged between 17% and 67%. The clinical characteristics of HF patients of different EF are represented in Table 1. As shown in Figure 4, there was a significant decrease in triglycerides and VLDL‐cholesterol as the EF decreases, whereas LDL‐cholesterol levels generally increased as EF decreases. Increased total cholesterol to HDL ratios were generally observed as the EF decreases, although this increase was not significant. No significant change was observed in total cholesterol and HDL levels.

### Correlation between plasma total cholesterol levels and Ox‐LDL in PB and LVB

**Figure 5:**
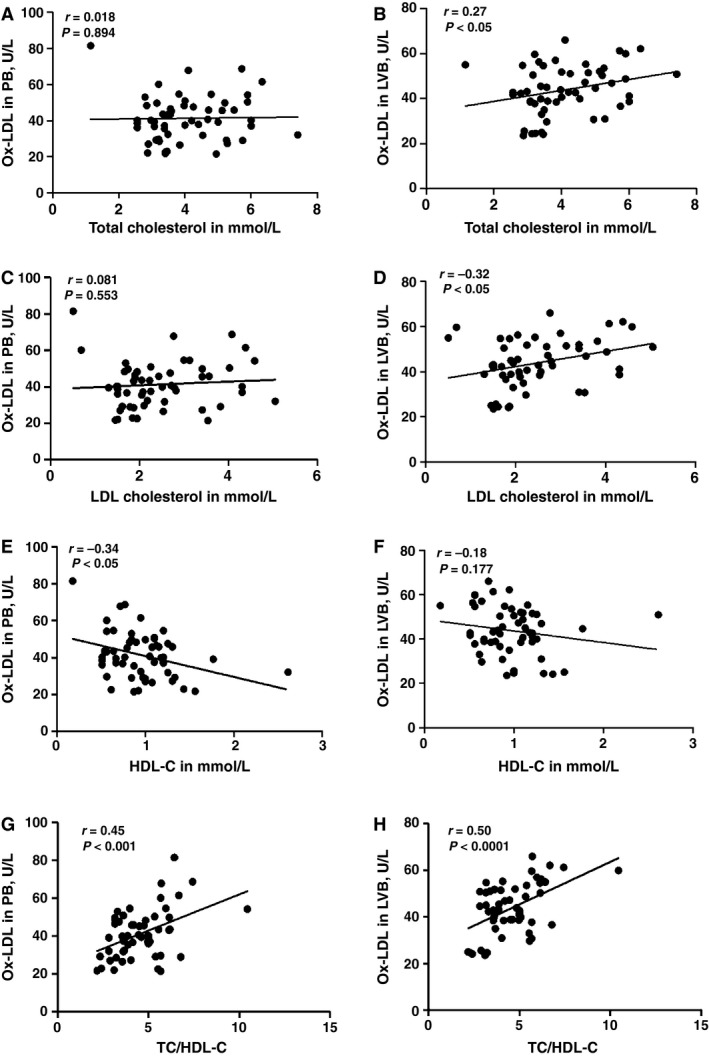
Correlation between plasma cholesterol and Ox‐LDL in PB and LVB: Correlation of Ox‐LDL in both PB and LVB were compared with plasma total cholesterol (A, B), LDL‐C (C,D), HDL‐C (E, F), and TC/HDL‐C ratio (G,H). Pearson correlation coefficient *r* is shown.(*P <* *0.05)

As shown in Figure 5, Ox‐LDL in both LVB and PB were compared with plasma total cholesterol, LDL‐C, HDL‐C, and TC/HDL‐C levels (A, C, and G). There was a positive correlation in LVB Ox‐LDL versus plasma total cholesterol, LDL‐C and TC/HDL‐C levels (B, D, and H), whereas negative correlation was observed in HDL‐C levels (E and F) of HF patients of all EFs. Pearson Correlation *P*‐value as well as *r* values are shown.

### Correlation between plasma LDL‐cholesterol levels and Ox‐LDL in PB and LVB of HF patients of all EFs

**Figure 6:**
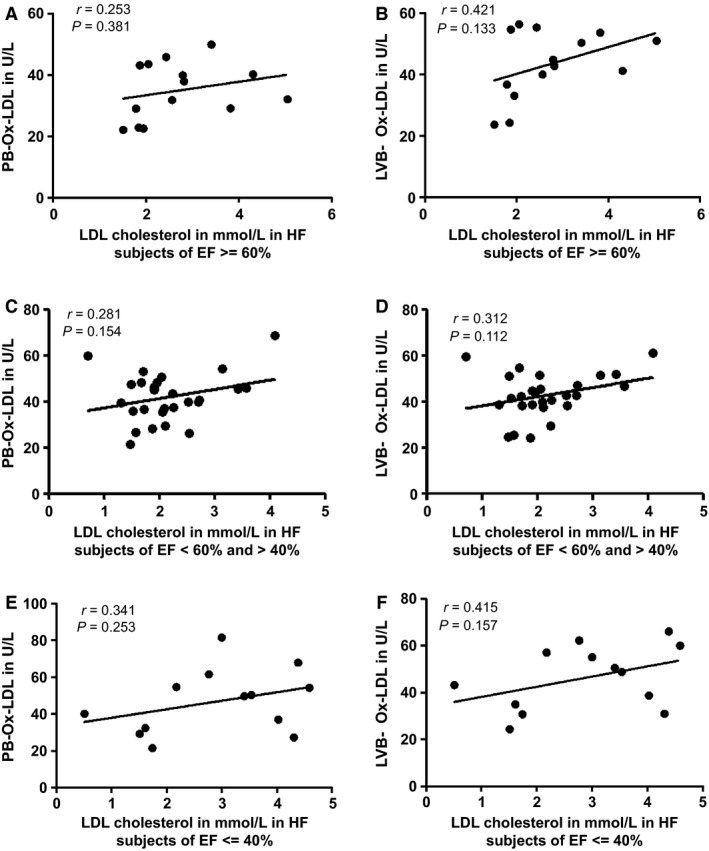
Correlation between plasma LDL‐cholesterol levels and Ox‐LDL in PB and LVB of HF patients of all EFs: Ox‐LDL levels in both LVB and PB were compared with plasma LDL‐C of HF patients of all the EFs. Pearson Correlation *P*‐value as well as *r* values are shown.

As shown in Figure 6, Ox‐LDL levels in both LVB and PB were compared with plasma LDL‐C levels. There was a positive correlation in both blood sites for patients of all EFs. Pearson Correlation *P*‐value as well as *r* values are shown.

### Troponin levels

**Figure 7:**
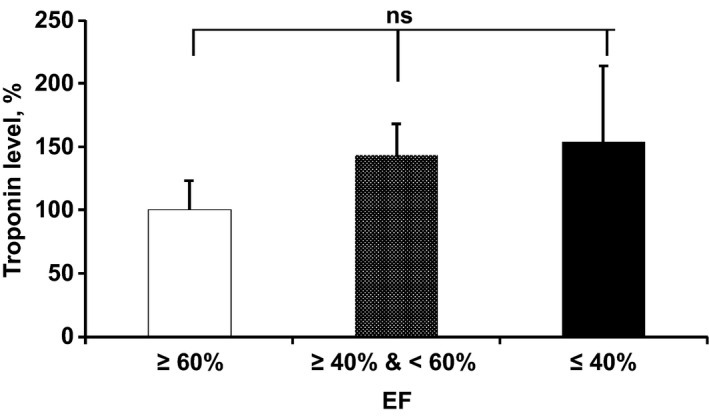
Plasma troponin levels of HF patients. Levels of troponin in HF subjects with EF >40% and <60% and EF ≤ 40% is expressed as a percentage compared to the level in HF patients with EF ≥ 60%. Values for each EF group are expressed as mean ± SD.

Increased troponin levels were observed in patients with lower EF. As shown in Figure 7, troponin levels increased with decreasing EF. The relationship between the degree of elevation of cardiac troponin I and the EFs was assessed by using the following categories: EF ≤ 40% (0.7 *μ*g/mL); EF >40% and <60% (0.57 *μ*g/mL); and EF ≥ 60% (0.32 *μ*g/mL). As the EF reduced, troponin levels increased in EF >40% and <60%, and EF ≤ 40% 43% and 54%, respectively, as compared to EF ≥ 60%.

### Quantitative measurement of cholesterol efflux from foam cells in presence of HF plasma samples

**Figure 8:**
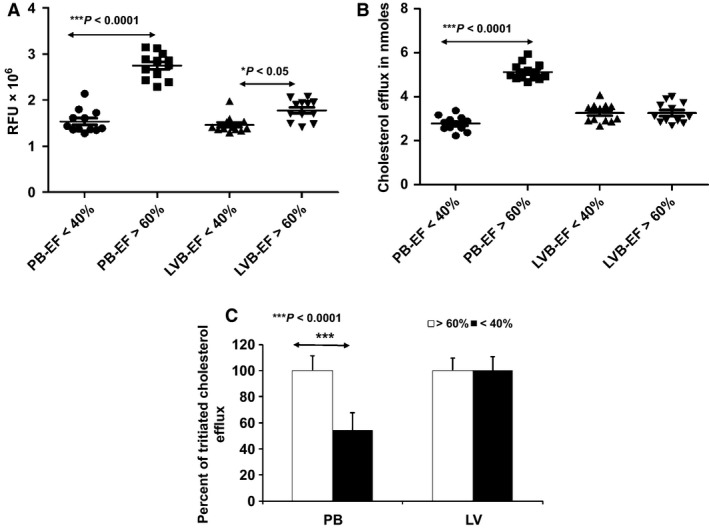
Cholesterol efflux capacity of HF samples: PB and LVB plasma samples from subjects with lower and normal EF were incubated with foam cells loaded with (A) NBD‐cholesterol and the efflux of NBD‐cholesterol was measured following deproteination of the samples. (B) Similarly, PB and LVB plasma samples from the lower and normal EFs were incubated with ^3^H cholesterol containing foam cells and the efflux of ^3^H cholesterol was measured followed by conversion of CPM value to nmoles of effluxed cholesterol. Cholesterol efflux associated with PB plasma from subjects with normal EF was compared with subjects of low EF. Similarly, cholesterol efflux associated with the LVB plasma from the two groups were analyzed. (C) Percent efflux from tritiated cholesterol loaded macrophages. All the samples were analyzed in triplicates and values are expressed as mean ± SD (*n* ≥ 3) *P < 0.05 and ***P < 0.0001 (One‐way ANOVA with Bonferroni's multiple comparison test).

Foam cells were developed by incubating RAW 264.7 macrophages with cholesterol (NBD‐cholesterol or H^3^ cholesterol)/LysoPC mixed micelles and used for the efflux study in the presence of HF plasma samples. Foam cells were incubated with 5 *μ*L of plasma samples. After 4 h, the medium was used to measure the presence of NBD‐cholesterol. As shown in Figure 8A, increased cholesterol efflux was observed in PB of HF subjects with EF > 60%, as compared to LV blood. There was a significant difference between cholesterol efflux of the PB and LV blood of HF subjects of EF > 60%, whereas no such difference was observed with the subjects of EF < 40%. Interestingly, cholesterol efflux was similar in both LVB and PB samples of patients with EF < 40%.

These results were further validated by using ^3^H‐cholesterol. The counts per minute (CPM) value for ^3^H‐cholesterol efflux was converted into nmoles. As shown in Figure 8B, an increased amount of cholesterol efflux was (~5–6 nmol) observed in the PB of subjects with EF > 60% as compared to matched LV blood (~1.5–2 nmol). These results corroborated our findings from the fluorescence cholesterol efflux studies. The percent of cholesterol efflux was represented in Figure 8C. A significant reduction (approximately 46%) in cholesterol efflux was observed in PB samples of patients with ≤40% EF as compared to that of patients with EF ≥ 60%. These cholesterol efflux studies correlate with PON1 activity of the same samples. The correlation between cholesterol effluxes and PON1 activity have been presented in Figure S1. Pearson Correlation *P*‐values, as well as *r* values, are shown.

## Discussion

This study is the first to demonstrate that Ox‐LDL might be present in LV blood in significantly higher amounts as compared to levels in peripheral blood when the EF is near normal. However, there was no difference observed in LVB as EF reduces in HF patients, whereas increased amounts of Ox‐LDL was observed in PB. As shown in Figure 2, we have demonstrated elevated levels of Ox‐LDL in the LV cavity and PB in subjects with depressed EF. Elective CABG with normal EF could be used as controls; our primary objective was to demonstrate elevated levels of Ox‐LDL in LV cavity in subjects with impaired EF considering normal EF subjects as controls. We and others have reported an influx of leukocytes in infarcted LV tissue during the development of HF (Kawakami et al. 2004; Chandrakala et al. 2013). Both monocytes and neutrophils secrete myeloperoxidase (MPO), an oxidative enzyme. Whether MPO secreted in the extracellular fluid, or peroxidized lipids formed on the myocyte cell membrane during ischemia/reperfusion injury, subsequently transferred to LDL in the left ventricle, could be the cause of Ox‐LDL generation can only be speculated. As Ox‐LDL has been reported to be increased in many risk factors associated with HF, it is likely that in addition to direct myocardial oxidative injury, systemic elevation of OX‐LDL also could contribute to the induction of brain natriuretic peptide (BNP) (Chandrakala et al. 2012) and other oxidative stress responsive genes in the heart tissue. However, the increased presence of Ox‐LDL in the left ventricular blood might suggest that localized formation of Ox‐LDL could also be an important contributor.

Existing evidence suggests that Ox‐LDL will be cleared quickly by the liver; however, under oxidative stress conditions, end organ dysfunction could affect clearance mechanisms which, in turn, would lead to increased levels of Ox‐LDL in the peripheral blood. Yet another possibility is that reduced paraoxonase activity might also be the cause of increased Ox‐LDL levels. To clarify this possibility, we compared PON1 activity in both blood samples of HF patients.

The results of many studies, in vitro and in vivo*,* suggest that the activity or concentration of PON1 is inversely related to atherosclerotic processes, in which Ox‐LDL plays a pivotal role. In this study, we observed a similar association between circulating Ox‐LDL and paraoxonase activity of PON1 in HF patients. The cardioprotective role of HDL is largely due to the PON1 located on it (Mackness et al. 1993; Mackness 1994; Watson et al. 1995; Graham et al. 1997; Aviram et al. 1999). PON1 metabolizes mildly oxidized phospholipids, presumably by eliminating hydroperoxy derivatives of unsaturated fatty acids (Watson et al. 1995). This effect of HDL in decreasing LDL lipid peroxidation is maintained for longer than that of antioxidant vitamins and could therefore be more protective. In addition, the two other members of the PON gene family, PON2 and PON3, may also have important antioxidant properties. Thus, the PON1‐CVD association is expected to result from the role of PON1 in the metabolism of bioactive lipid molecules and protection against damage due to oxidized LDL. It has been identified that subjects of coronary heart disease (CHD) have low serum PON1 activity. Existing studies also support low serum PON1 activity as an independent predictor of new CHD events. In our studies, paraoxonase levels were reduced both in LVB and PB plasma samples of subjects with EF ≤ 40%, suggesting that the reduced blood supply and oxygen to the end organs might also contribute to oxidative stress and dysfunction (Fig. 3).

During heart failure, accumulation of reactive oxygen species could decrease paraoxonase activity by modifying the synthesis or secretion or by directly inhibiting the enzyme (Sunil et al. 2013). A reduction in serum PON1 activity, as seen in the study, may increase the oxidative stress and makes not only LDL more susceptible to oxidation, but all other serum lipoproteins, including HDL. HDL oxidation may reduce its functionality to induce cellular cholesterol efflux from macrophages. Hence, rather than the absolute levels of HDL, the quality of HDL is maintained by its protective enzyme PON1 and is altered in several pathological conditions such as obesity, myocardial infarction, dyslipidemia and diabetes (Mackness et al. 1993; Watson et al. 1995). The decrease in PON1 activity in subjects of low ejection fraction suggests a degree of liver damage by oxidative stress. This reduced PON1 activity might be due to defective gene expression in the liver, but could also result from altered synthesis and/or secretion of HDL. Altered secretion of HDL often results from impaired lecithin:cholesterol acyl transferase (LCAT) activity. Impaired LCAT activity is also associated with several other oxidative stress‐related diseases, such as cirrhosis, chronic liver disease, and type 2 diabetes, among others.

The independent association of elevated triglycerides to the risk of future CVD events has long been controversial. Our studies indicate the opposite of the common expectation of increased triglycerides correlated to increased CVD risk. As shown in Figure 4, TRG levels were significantly decreased in plasma samples of subjects with EF ≤ 40% as compared to subjects with EF ≥ 60%. In addition, VLDL levels are also significantly reduced in patients with EF ≤ 40%. Hepatic production of triglycerides is coupled to that of ApoB‐100 to form VLDL. The reduced TRG and VLDL levels in subjects with ≤40% EF might due to end organ (liver) dysfunction, leading to reduced lipolysis or secretion caused by oxidative stress. Yet another possibility is that the higher TRG levels in the EF ≥ 60% group is caused by a comorbidity, such as diabetes.

Foam cells developed by using fluorescent and tritiated cholesterol/LysoPC mixed micelles were efficiently used to study cholesterol efflux by HF patients. Various studies have suggested oxidation of HDL leads to the loss of its antiatherogenic property. Dysfunctionality of HDL has attained more attention in the last two decades. Several researchers have been working on identifying the factors responsible for drastic modifications of structure and composition of HDL and to address the outcomes associated with HDL dysfunctionality. Acute‐phase response, inflammation, and oxidative stress render HDL dysfunctional with highly adverse consequences. During acute phase response and oxidative stress, modification of amino acids in Apolipoprotein A1 (APOA1) and inactivation of HDL‐associated enzymes, such as PON1, leads to the dysfunction of HDL. In addition, changes in the lipid composition of HDL might also contribute significantly to this phenomenon.

As we expected, low cholesterol efflux was observed in plasma samples (both LVB and PB) of HF patients of EF < 40%, and LVB of patients of EF > 60% as compared to PB of HF subjects with EF > 60% is probably due to the oxidized HDL formed by oxidative stress. This observation leads us to an important question that the use of PB alone to determine the CE and HDL quality/functionality may not be enough to predict CVD in an individual. Low HDL‐cholesterol levels are associated with coronary heart disease, but in our study, we did not find a difference in HDL levels between subjects with higher and lower EF. However, when the cholesterol efflux capacity and PON1 activity associated with HDL were compared, it became clear that patients with normal EF have a higher amount of functional HDL than that found in the patients with low EF. From this observation, it can be concluded that the measurement of cholesterol efflux capacity or PON1 activity is a much better indicator of HDL quality and functionality than the concentration of HDL alone. Together with an increased ratio of total cholesterol:HDL‐cholesterol, the data suggest a decrease in reverse cholesterol transport. This might be due to the dysfunctionality of HDL which results in reduced cholesterol efflux, decreased oxidative protection of LDL by HDL or increased cholesterol influx by macrophages. In conclusion, dysfunctional HDL increases the risk of atherosclerosis and cardiovascular disease by several‐fold as opposed to the atheroprotective functions of native HDL.

## Conflict of Interest

None declared.

## Supporting information




**Figure S1.** Correlation between cholesterol efflux and PON1 activity: Correlation between PON1 activity and cholesterol efflux were compared of the EFs ≥60% and ≤40%. Pearson Correlation *P*‐value as well as *r* values are shown.

**Table S1.** Measurement characteristics of Simpson biplane method.
