# Supplementary figures and images for: Increased presence of oxidized low‐density lipoprotein in the left ventricular blood of subjects with cardiovascular disease
Source: Physiol Rep. 2016 Mar 31;4(6):e12726. doi: 10.14814/phy2.12726 (PMC4814879; doi:10.14814/phy2.12726)

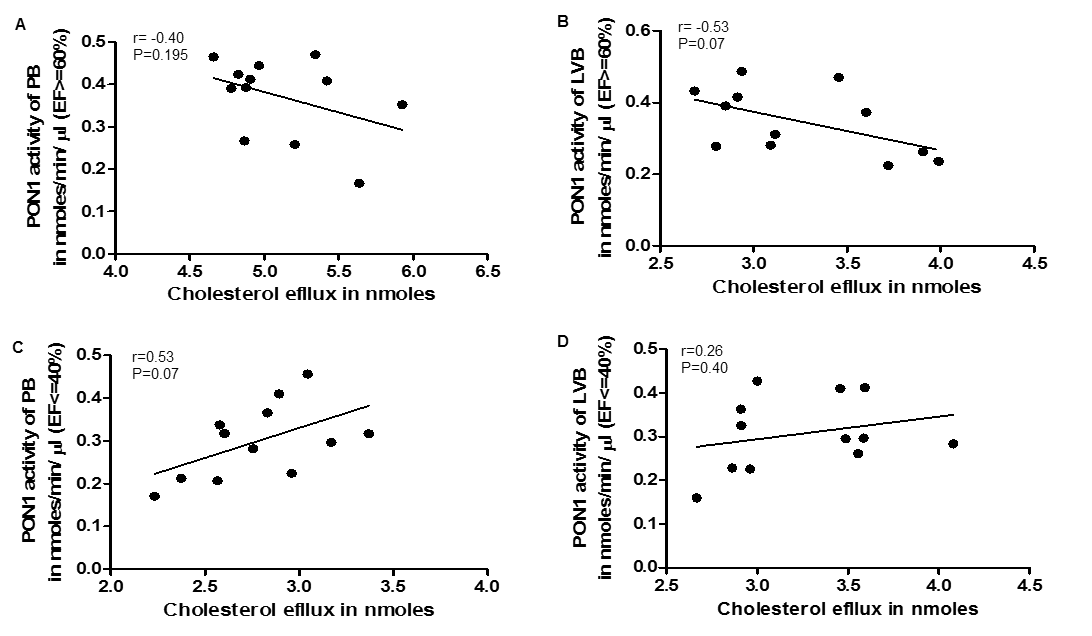

Supplement: Supplementary file 1 — Figure S1. Correlation between cholesterol efflux and PON1 activity: Correlation between PON1 activity and cholesterol efflux were compared of the EFs ≥60% and ≤40%. Pearson Correlation P‐value as well as r values are shown. [file PHY2-4-e12726-s001.tif]
