# Supplementary material for: Increased presence of oxidized low‐density lipoprotein in the left ventricular blood of subjects with cardiovascular disease
Source: Physiol Rep. 2016 Mar 31;4(6):e12726. doi: 10.14814/phy2.12726 (PMC4814879; doi:10.14814/phy2.12726)
Supplement: Supplementary file 2 — Table S1. Measurement characteristics of Simpson biplane method. [file PHY2-4-e12726-s002.docx]

**Supplementary Table 1: Measurement characteristics of Simpson biplane method**

|  |  |  |
| --- | --- | --- |
|  | Original EF | Considered  for the study |
| Normal | >55% | ≥ 60% |
| Mild | 45-54% | >40% and <60% |
| Moderate | 30-44% |  |
| Severe | <30% | ≤40% |

±5% added to original EF and considered for the study.
